# MGA-YOLO: A lightweight one-stage network for apple leaf disease detection

**DOI:** 10.3389/fpls.2022.927424

**Published:** 2022-08-22

**Authors:** Yiwen Wang, Yaojun Wang, Jingbo Zhao

**Affiliations:** College of Information and Electrical Engineering, China Agricultural University, Beijing, China

**Keywords:** apple leaf disease, object detection, MGA-YOLO, attention mechanism, CBAM, lightweight CNN

## Abstract

Apple leaf diseases seriously damage the yield and quality of apples. Current apple leaf disease diagnosis methods primarily rely on human visual inspection, which often results in low efficiency and insufficient accuracy. Many computer vision algorithms have been proposed to diagnose apple leaf diseases, but most of them are designed to run on high-performance GPUs. This potentially limits their application in the field, in which mobile devices are expected to be used to perform computer vision-based disease diagnosis on the spot. In this paper, we propose a lightweight one-stage network, called the Mobile Ghost Attention YOLO network (MGA-YOLO), which enables real-time diagnosis of apple leaf diseases on mobile devices. We also built a dataset, called the Apple Leaf Disease Object Detection dataset (ALDOD), that contains 8,838 images of healthy and infected apple leaves with complex backgrounds, collected from existing public datasets. In our proposed model, we replaced the ordinary convolution with the Ghost module to significantly reduce the number of parameters and floating point operations (FLOPs) due to cheap operations of the Ghost module. We then constructed the Mobile Inverted Residual Bottleneck Convolution and integrated the Convolutional Block Attention Module (CBAM) into the YOLO network to improve its performance on feature extraction. Finally, an extra prediction head was added to detect extra large objects. We tested our method on the ALDOD testing set. Results showed that our method outperformed other state-of-the-art methods with the highest *mAP* of 89.3%, the smallest model size of only 10.34 MB and the highest frames per second (FPS) of 84.1 on the GPU server. The proposed model was also tested on a mobile phone, which achieved 12.5 FPS. In addition, by applying image augmentation techniques on the dataset, *mAP* of our method was further improved to 94.0%. These results suggest that our model can accurately and efficiently detect apple leaf diseases and can be used for real-time detection of apple leaf diseases on mobile devices.

## 1. Introduction

Apple is one of the most important economic fruits in the world. However, various apple leaf diseases pose great threats to the productivity and the quality of apples, causing significant economic losses. Given available methods for diagnosis, apple leaf disease management still faces great challenges. At present, apple leaf disease diagnosis primarily relies on visual inspection by trained experts (Liu et al., 2017). As the method is subjective, it often leads to misdiagnosis, resulting in low efficiency and insufficient accuracy.

The development of computer hardware and software technology has enabled agriculture and computer engineering technology to be more closely linked. With the help of innovative tools, such as computer vision, machine learning, and deep learning algorithms, smart agriculture applications are flourishing. Such applications typically include precision agriculture, disease diagnosis, and crop phenotyping (Pathan et al., 2020). As frontiers of artificial intelligence, machine learning algorithms are being progressively used in crop leaf disease diagnosis. However, classical machine learning algorithms mostly rely on hand-crafted low-level vision features, which are designed based on engineering experience. This often results in unsatisfactory performance when the captured scene is comparatively complex.

Recently, deep learning models, such as convolutional neural networks (CNNs), have made great progress compared with classical machine learning methods. CNN-based models provide end-to-end pipelines to automatically learn low-level discriminative features and model parameters, making it easier for non-experts to tackle computer vision-based tasks of crop disease diagnosis (Liu et al., 2017; Sun et al., 2021). However, due to abundant parameters and the high computational overhead of CNNs, most CNN models for apple leaf disease diagnosis are implemented on high-performance servers with GPU acceleration. This limits their application in the field (Agarwal et al., 2020). To make CNN models more practical and suited for deployment on mobile devices for real-time detection, many lightweight CNNs have been proposed. Lightweight models reduce the number of parameters, but leads to a slight decline in accuracy. To compensate for the accuracy loss of lightweight models, attention mechanisms can be used to distribute different weights to each part of the input feature layers, extract essential features, and improve classification performance. In addition, attention mechanisms have little impact on efficiency and do not require large storage space for the model (Wang et al., 2021).

Image classification using CNN models has been widely used in apple leaf disease classification. However, image classification is insufficient for practical application scenarios as detailed information in an image needs to be obtained, including the number and regions of infected leaves. In this case, Object detection is more useful for disease diagnosis. Current object detection methods based on deep learning algorithms include one-stage object detection algorithms and two-stage object detection algorithms. Representatives of one-stage algorithms are SSD (Liu et al., 2016), RetinaNet (Lin et al., 2017), YOLOv4 (Bochkovskiy et al., 2020), YOLOv5 (Jocher et al., 2021), DETR (Carion et al., 2020), FCOS (Tian et al., 2019), YOLOX (Ge et al., 2021). Two-stage algorithms include Faster-RCNN (Ren et al., 2016), Cascade-RCNN (Cai and Vasconcelos, 2018), etc. One-stage algorithms are more suited than two-stage algorithms for practical application scenarios as the speed of one-stage object detection algorithms is usually faster than two-stage object detection algorithms.

This paper proposes a novel CNN model for apple leaf disease recognition, tested on a new dataset built by us. The main contributions are two-fold:

(1) We propose a lightweight one-stage CNN model, called the Mobile Ghost with Attention YOLO network (MGA-YOLO), based on YOLOv5 for real-time apple leaf disease recognition. The Ghost module and the depthwise separable convolution are exploited to significantly reduce the number of parameters and FLOPs. The Mobile Inverted Residual Bottleneck Convolution that integrates CBAM is constructed to improve feature extraction capability. We also add a prediction head to detect extra large objects. The GELU non-linearity is utilized for optimal fine-tuning.
(2) We built a new dataset, called the Apple Leaf Disease Object Detection dataset (ALDOD), using images collected from two public datasets: Plant Pathology 2021-FGVC8 and Plant Pathology 2020-FGVC7 on Kaggle. These two datasets only contain labels for each image. Our contribution here is that we manually annotated each leaf in every image with a bounding box and a class label, which belongs to one of the four categories: healthy, rust, scab and black rot. This dataset will be made publicly available, which will further related research in apple leaf disease recognition.

## 2. Related work

### 2.1. Traditional machine learning methods

Traditional machine learning methods for apple leaf disease recognition usually consist of three steps: image pre-processing, feature extraction, and disease recognition. Firstly, images are pre-processed by converting them from the RGB color space to another color space, e.g., YUV and HSV. Contrast stretching and other methods are also often used to improve the quality of images. Backgrounds are removed in the pre-processing process. Then, texture and shape features are extracted by statistical methods, such as GLCM (Fulari et al., 2020; Jan and Ahmad, 2020) and KPCA. Finally, machine learning-based models, such as the Support Vector Machine (SVM), random forests, and decision trees (Zhang et al., 2020), are used as classifiers to identify crop leaf diseases. However, such methods are time-consuming and are unable to cope with complex image features, which result in unsatisfactory efficiency and accuracy.

### 2.2. Deep learning methods

Due to efficient structures of end-to-end pipelines and high classification accuracy, deep learning models have been progressively used in agriculture. Researchers have conducted many studies on apple leaf disease recognition using deep convolutional networks. Li and Rai (2020) compared the performance of the SVM, VGG, and ResNet, concluding that ResNet-18 achieved better recognition results. Jwo and Chiu (2022) proposed a model based on CNNs with 19 convolutional layers for effective and accurate classification of Marsonina Coronaria and Apple Scab diseases from apple leaves. Rehman et al. (2021) proposed a parallel framework for apple leaf disease recognition. The Mask RCNN was configured to detect the infected regions. Meanwhile, the augmented images were used to train a pre-trained CNN model based on ResNet50 to extract features of images for classification. CNN-based models achieved better performance of recognition for apple leaf diseases than traditional machine learning models (Li and Rai, 2020).

### 2.3. Lightweight CNNs

In practical application scenarios, mobile devices often do not have sufficient storage space to store considerable numbers of parameters in deep convolutional networks. Under this circumstance, researchers have proposed feasible methods. Sun et al. (2021) proposed a lightweight CNN model, called the MEAN-SSD, that can be deployed on mobile devices to detect apple leaf diseases in real-time. A basic module called the Mobile End AppleNet block was proposed to increase the detection speed and reduce the model's size by reconstructing the typical 3 × 3 convolution. Bi et al. (2022) proposed a stable, low-cost and high-precision apple leaf diseases recognition method by employing the MobileNet model to save detection time and improve efficiency. The DCNN model proposed by Chao et al. (2020) for apple leaf disease recognition combined the DenseNet and the Xception, using global average pooling instead of fully connected layers. These studies showed that lightweight CNN models have better performance in terms of detection speed and accuracy in mobile devices.

### 2.4. The attention mechanism

Attention is a cognitive process that acts selectively on relevant information while ignoring others in deep neural networks. Originated from Natural Language Processing (NLP), it has been widely used in computer vision techniques to extract essential features of the input data and ignore redundant information. Hu et al. (2018) proposed the Squeeze-and-Excitation (SE) block and won the best image classification champion of ImageNet 2017. Woo et al. (2018) proposed the Convolutional Block Attention Module (CBAM), which combined channel attention and spatial attention. Compared with the SE module, CBAM added a spatial attention mechanism to concentrate on the region of interest and can be integrated into any CNN architecture. In addition, their experiments showed significant improvements in classification and detection performance owing to the application of the CBAM. To alleviate the loss of location information caused by the 2D global pooling of the previous attention module, Hou et al. (2021) proposed the Coordinate Attention (CA) that decomposed the channel attention into two parallel 1-D feature encoding processes and effectively integrated the spatial coordinate information into the generated attention map. Existing research shows that the attention mechanism has a strong potential for apple leaf disease recognition. Yu and Son (2020) proposed a leaf spot attention network that had two sub-networks. The first was for feature segmentation to provide more discriminative features while the other was a spot-aware classification sub-network to identify apple leaf diseases. The architecture outperformed conventional state-of-the-art deep learning models. Wang et al. (2021) introduced a deep learning model with an attention mechanism, called the Coordination Attention EfficientNet, to identify different apple leaf diseases. The coordinate attention block was embedded into the novel deep convolutional neural network and extracted important channel features and spatial location information. The results of these experiments showed that attention mechanisms effectively improved the accuracy of apple leaf disease recognition.

## 3. Methods

In this section, we introduce MGA-YOLO in detail. MGA-YOLO is based on the YOLOv5 network. We used the Ghost module and constructed the Ghost bottleneck. The attention mechanism was introduced through the CBAM module. With CBAM embedded, the Mobile Inverted Residual Bottleneck Convolution was established. Improvements have also been made to the detection head and the non-linear activation functions in the whole network. We next explain each module of the network in detail.

### 3.1. Overview of the YOLOv5 network

YOLO, which stands for “You Only Look Once,” is an object detection algorithm with excellent accuracy and detection speed. YOLOv5 utilizes depth and width multiples to scale the depth of the network and the number of convolution kernel channels in each layer. YOLOv5 has four versions, including YOLOv5s, YOLOv5m, YOLOv5l, and YOLOv5x, whose depth and width increase in series. YOLOv5s is the simplest version with the smallest number of network parameters and the fastest inference speed.

In general, the YOLOv5 network is divided into three components: the backbone network, the neck network, and the head. The architecture of the backbone network is CSPDarknet with Focus, Conv-BN-LeakyReLU (CBL), BottleneckCSP_X, and the Spatial Pyramid Pooling (SPP) layer. The backbone network extracts image features and then the feature maps are transferred to the neck network for feature enhancement. The neck network aggregates low-level spatial features and high-level semantic features through the Path Aggregation Network (PANet). It significantly improves the accuracy to identify objects of different scales. Finally, the head generates object bounding boxes with coordinates, categories, and confidence.

**Figure 1:**
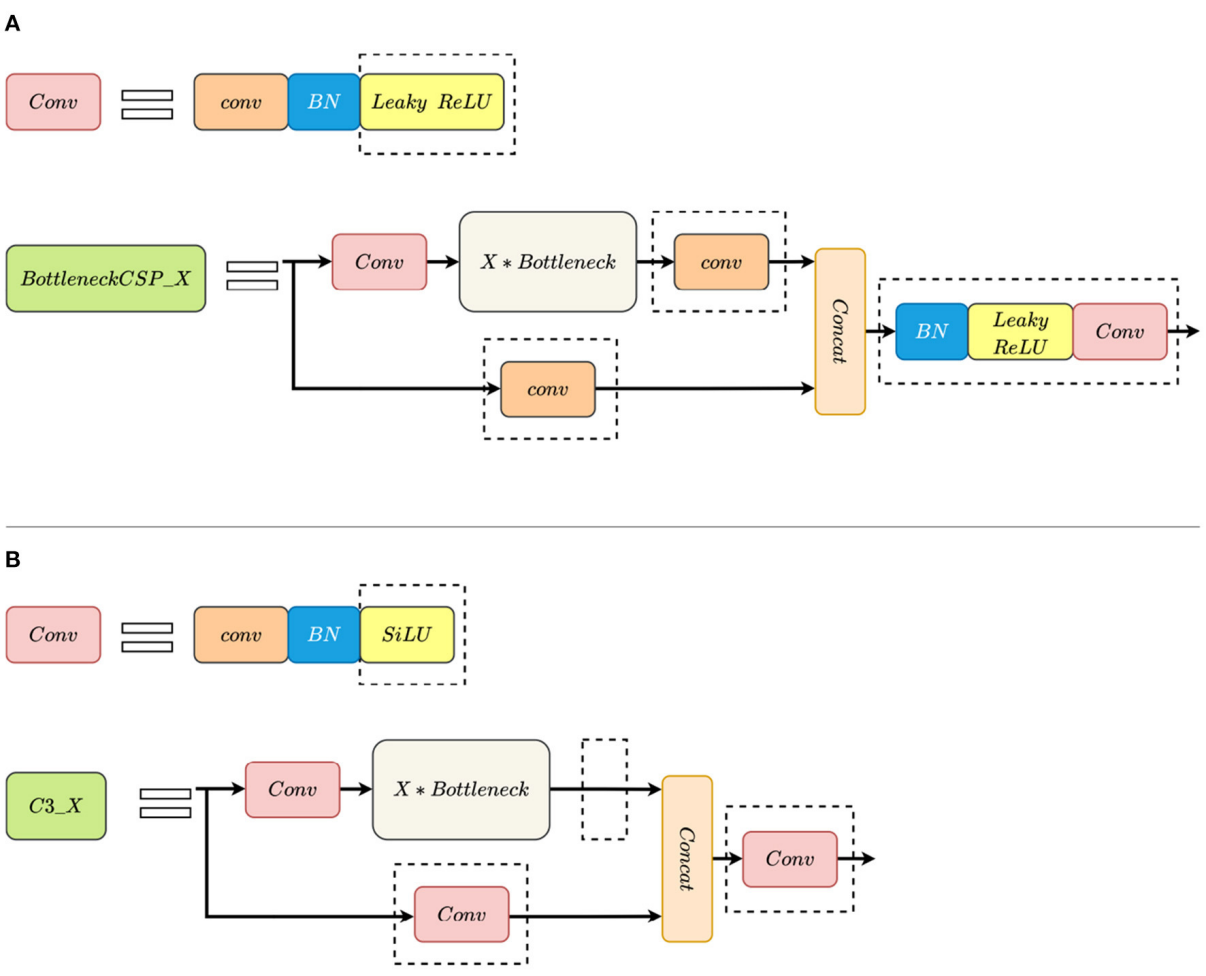
Comparison between BottleneckCSP_X and C3_X modules. **(A)** BottleneckCSP_X. **(B)** C3_X. The dotted rectangle indicates the difference between them. SiLU replaces leaky ReLU. C3_X removes Conv after Bottleneck and replaces Conv in another path with Conv. Additionally, it abandons BN and Leaky ReLU after concatenating the output data from two paths.

Compared with YOLOv5 version 3.0, the structure of the CSP module is modified and the LeakyReLU activation function is replaced with the SiLU (Swish-1) activation function in YOLOv5 version 5.0. The modified BottleneckCSP is called C3_X. The convolution module and the CSP module changes are shown in Figure 1.

The YOLOv5 model is divided into YOLOv5-P5 and YOLOv5-P6 families. Each family includes models of different sizes. The size of the image input into the YOLOv5-P5 model is 640 × 640 pixels. YOLOv5-P5 models have three detection layers P3, P4, and P5 at strides of 8, 16, and 32, which are used to detect small, medium, and large objects. YOLOv5-P6 adds the P6 output layer at a stride of 64 intended for extra large objects. Correspondingly, the backbone is extended to P6, and the PANet neck goes down to P3 and back up to P6 instead of stopping at P5. P6 models increase performance on COCO especially on the higher resolution images, e.g., images with width and height of both 1,280 pixels. The added P6 prediction head improves *mAP* while correspondingly brings inference speed loss and more parameters.

### 3.2. The ghost module

To enable real-time apple leaf disease detection on embedded devices, we reduced model parameters and FLOPs by replacing convolutions with the Ghost module in the backbone network. As discussed by Han et al. (2020), the Ghost module generates more feature maps through cheap operations. The forward propagation process of the Ghost module can be divided into three parts. In the first part, it produces a handful of intrinsic feature maps by ordinary convolution filters. Then, ghost feature maps are generated by a series of cheap operations on each intrinsic feature. Lastly, the intrinsic feature maps obtained in the first part and the ghost feature maps in the second part are concatenated as the output.

The operation of an ordinary convolutional layer can be formulated as:


(1) $$\begin{array}{r} Y=X*f+b \end{array}$$

where * is the convolution operation, *b* is a bias term, *X* ∈ ℝ ^*c*×*h*×*w*^ are the input feature maps (*c* denotes the number of input channels, and *h* and *w* denote the height and width of the input maps, respectively), *Y* ∈ ℝ ^*h*′×*w*′×*n*^ are the output feature maps with *n* channels, and *f* ∈ ℝ^*c*×*k*×*k*×*n*^ are the convolution filter in convolutional layers. In addition, *h*′ and *w*′ are the height and the width of the output feature maps, and *k* × *k* is the kernel size of convolution filters *f*. The number of FLOPs can be computed as *h*′ · *w*′ · *k* · *k* · *c* · *n* when the output feature maps are all generated by convolutional operations. If the channel number *c* and the number of filters *n* are both large, considerable numbers of FLOPs will deplete the memory and the computational resources of mobile devices.

**Figure 2:**
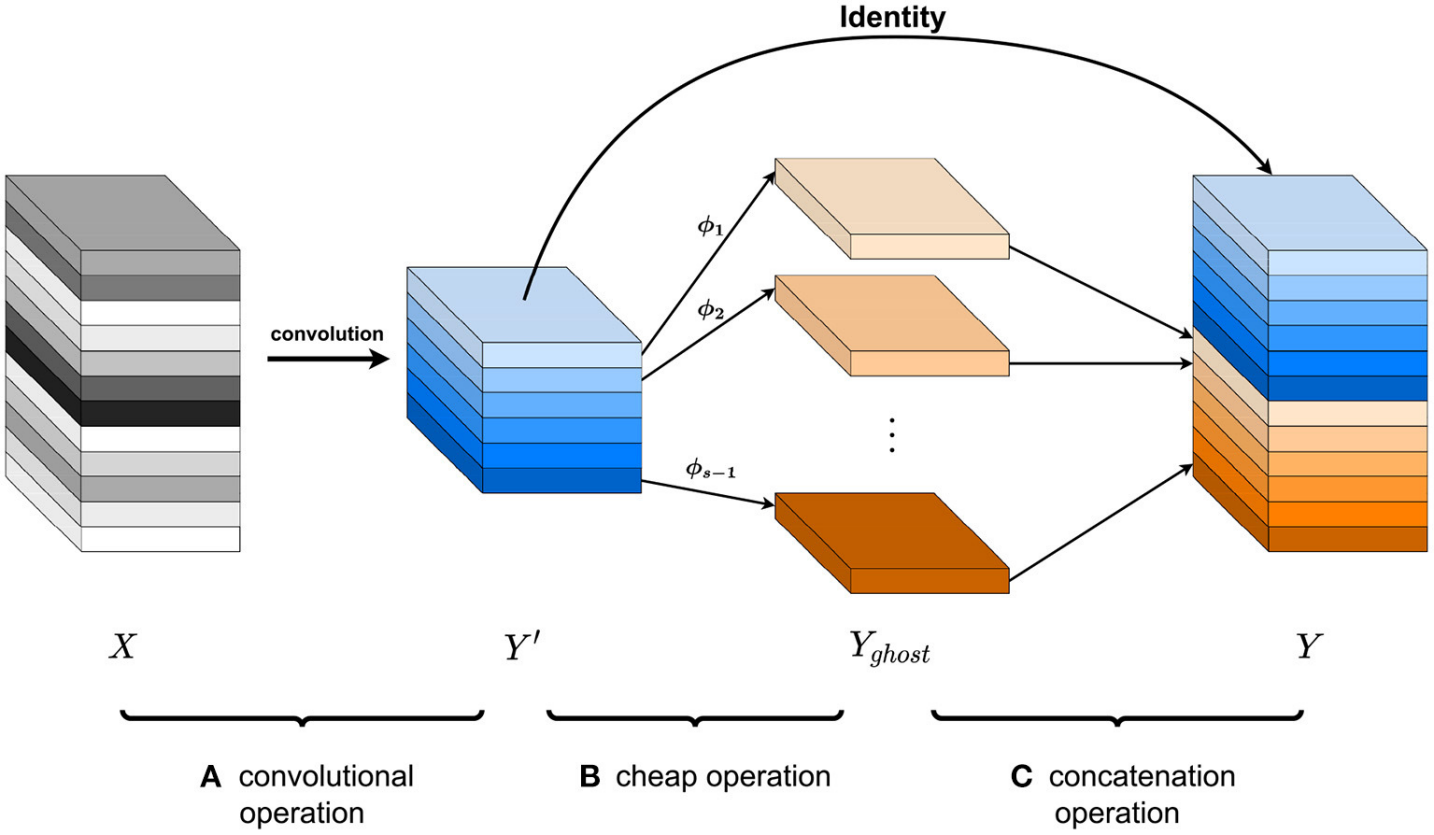
Three parts of Ghost module operation. **(A)** A convolutional operation generates intrinsic feature maps of *m* channels from the input maps of *n* channels. **(B)** The cheap operation generates the ghost feature maps of *s*−1 channels. **(C)** Concatenate *Y*′ and *Y*_*ghost*_, and *n* (*n* = *m*·*s*) output feature maps can be obtained.

The computational process of the ghost module can be expressed as:


(2) $$\begin{array}{r} Y^{\prime }=X*f^{\prime } \end{array}$$

(3) $$\begin{array}{r} Y_{ghost}=\phi_{j}(Y_{i}{}^{\prime }),j\in [1,s-1] \end{array}$$

where *Y*′ ∈ ℝ^*h*′×*w*′×*m*^ denote the output feature maps obtained by convolutional operations using filters *f*′ ∈ ℝ^*c*×*k*×*k*×*m*^ on the input feature layer *X* ∈ ℝ ^*c*×*h*×*w*^, *m* ≤ *n*. The bias term is omitted for simplicity. The number of FLOPs calculated by Equation (2) is *h*′ · *w*′ · *k* · *k* · *c* · *m*. In addition, $Y_{i}{}^{\prime }$ is the *i*-th feature map, and ***ϕ***_*j*_ is the *j*-th linear operation to generate the *j*-th ghost feature map, which means $Y_{i}{}^{\prime }$ generates *s*−1 ghost feature maps. Therefore, by Equation (3), we can obtain $Y_{ghost}\in {\mathbb{R}}^{h^{\prime }\times w^{\prime }\times \left[m\cdot \left(s-1\right)\right]}$. Finally, we obtain *n* (*n* = *m*·(*s*−1)+*m* = *m*·*s*) output feature maps *Y* by the concatenation operation:


(4) $$\begin{array}{r} Y=Y_{ghost}+Y^{\prime } \end{array}$$

where + is the concatenation operation for *Y*_*ghost*_ and *Y*′. The three parts of Ghost module operation is shown in Figure 2.

**Figure 3:**
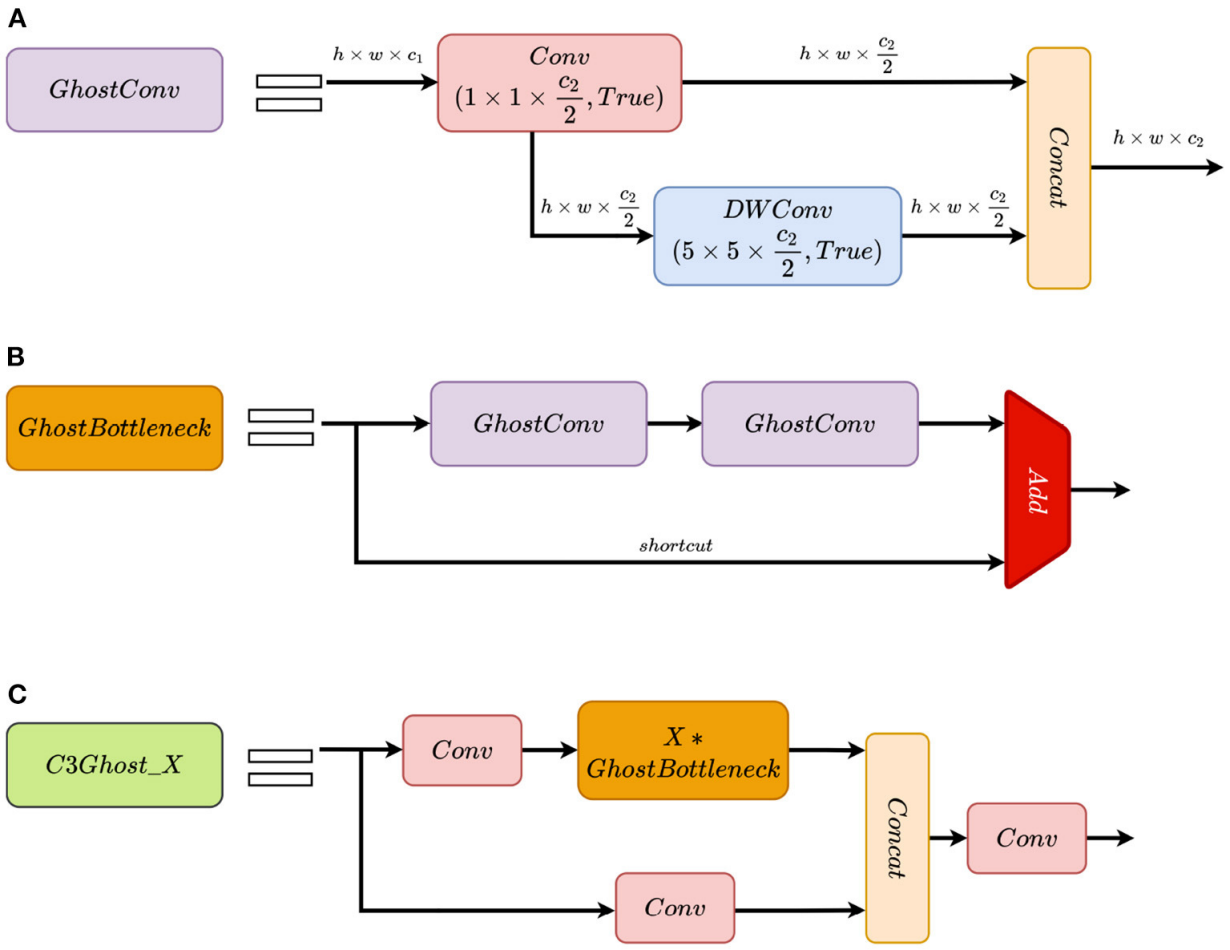
Structures of the Ghost module, the Ghost Bottleneck module, and the C3Ghost_X module. **(A)** GhostConv represents the Ghost module. The *h* × *w* × *c*_1_ data are first given to the 1 × 1 × *c*_2_/2 Conv module as an input to generate $\frac{h\times w\times c_{2}}{2}$ feature maps, and then sent to 5 × 5 × *c*_2_/2 DWConv module to obtain $\frac{h\times w\times c_{2}}{2}$ feature maps. Finally, the output feature maps from Conv and DWConv are concatenated to obtain *h* × *w* × *c*_2_ outputs. “True” indicates activation function is enabled. **(B)** GhostBottleneck is a residual block with two stacks of GhostConv and an identity shortcut from input. The output features are sum of the data from two paths above. **(C)** C3Ghost_X is the counterpart of C3_X module. Except that GhostBottleneck replaces Bottleneck, other parts are the same as C3_X.

The theoretical speed-up ratio of replacing ordinary convolutions with the Ghost module is approximately *s* (Han et al., 2020). Notably, the Ghost module has fewer computational parameters and FLOPs than the ordinary convolution layer. The Ghost Bottleneck module and the C3Ghost_X module are built based on the Ghost module. The Ghost Bottleneck mainly consists of two stacks of the Ghost modules. Structures of the Ghost module, the Ghost Bottleneck module and the C3Ghost_X module are shown in Figure 3. In the backbone network, we used the Ghost module with a stride of 2 for downsampling and used the C3Ghost_X module to extract image features instead of the ordinary convolution and the C3_X module. On the ALDOD training set, the Ghost modules increase convergence speed of training and the detection speed with almost no loss of the detection accuracy.

### 3.3. The convolutional block attention module (CBAM)

**Figure 4:**
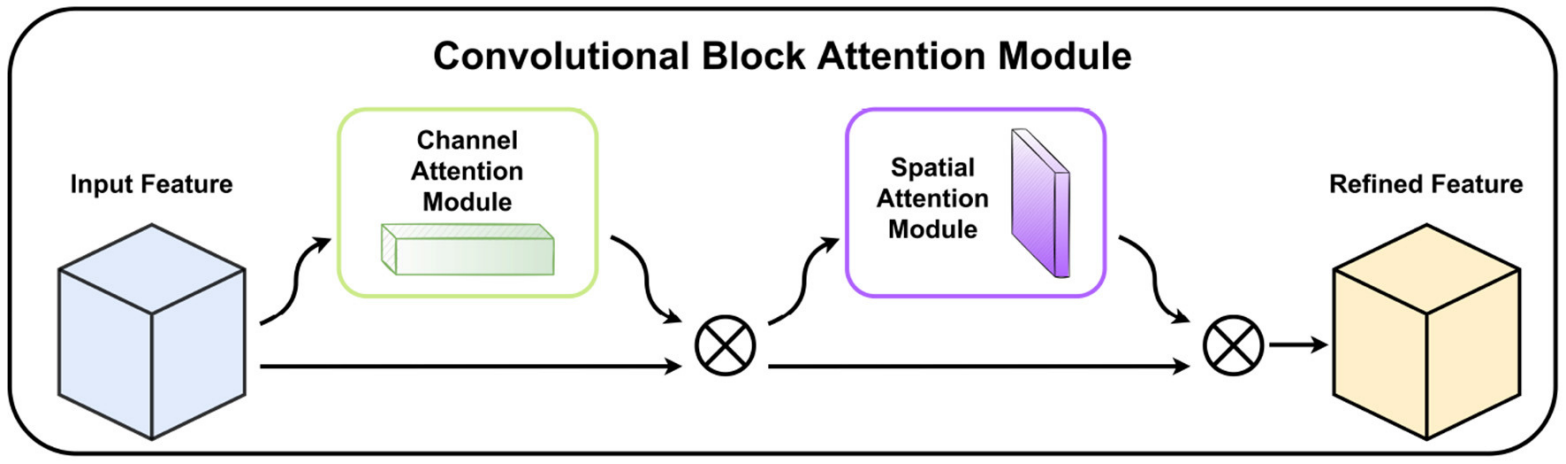
The structure of the Convolutional block attention module. Channel Attention Module and Spatial Attention Module sequentially refine feature maps. ⊗ denotes the multiplication of the input feature maps by the corresponding attention module.

CBAM (Woo et al., 2018) is a simple but effective attention module for feed-forward CNNs. CBAM contains two sequential sub-modules, called the Channel Attention Module and the Spatial Attention Module, which are applied in a particular order. When the feature maps are given as inputs, the module sequentially generates attention maps along channel and spatial dimensions, then the input feature maps are multiplied by the attention maps to get subsequent refined feature maps. Most importantly, CBAM is a lightweight module that can be trained end-to-end and can be smoothly embedded into any convolutional neural network. The structure of CBAM is shown in Figure 4.

Unlike the images with plain backgrounds, the images on ALDOD are mixed with complex background noise that interferes with feature extraction. Therefore, we integrated CBAM into our mobile-end network to concentrate on learning leaf spots features with little overhead.

### 3.4. Mobile inverted residual bottleneck convolution with attention mechanisms

Another important design in our network is the inverted residual linear bottleneck. MobileNetV2 (Sandler et al., 2018) demonstrated the superiority of Inverted Residuals and Linear Bottlenecks. In contrast with classical residuals, the inverted residuals in MobileNetV2 utilize 1 × 1 convolutional layers to expand channels of input features with an expansion ratio of 6, then the intermediate expansion layers use 3 × 3 depthwise convolutions to acquire non-linearities, finally 1 × 1 linear convolutional layers are used to reduce dimensions. Additionally, it uses shortcuts directly between the bottlenecks. It is shown that the inverted residual structure can compress the model parameters as much as possible with a small reduction in accuracy and the linear layers are capable of preventing excessive information loss when high-dimensional information is projected to low-dimensional information. MobileNetV3 (Howard et al., 2019) attaches the Squeeze-and-Excitation (SE) (Hu et al., 2018) module after the expansion layers for channel attention extraction. Inspired by MobileNetV2 and MobileNetV3, we maintained the general structure of the inverted residual linear bottleneck and integrate CBAM into it as a replacement for SE, adding the capability to extract spatial information. In addition, the ReLU or the h-swish (Howard et al., 2019) activation function in the first 1 × 1 convolution, which is used in MobileNet, is substituted by GELU non-linearity (introduced in Section 3.5).

**Figure 5:**
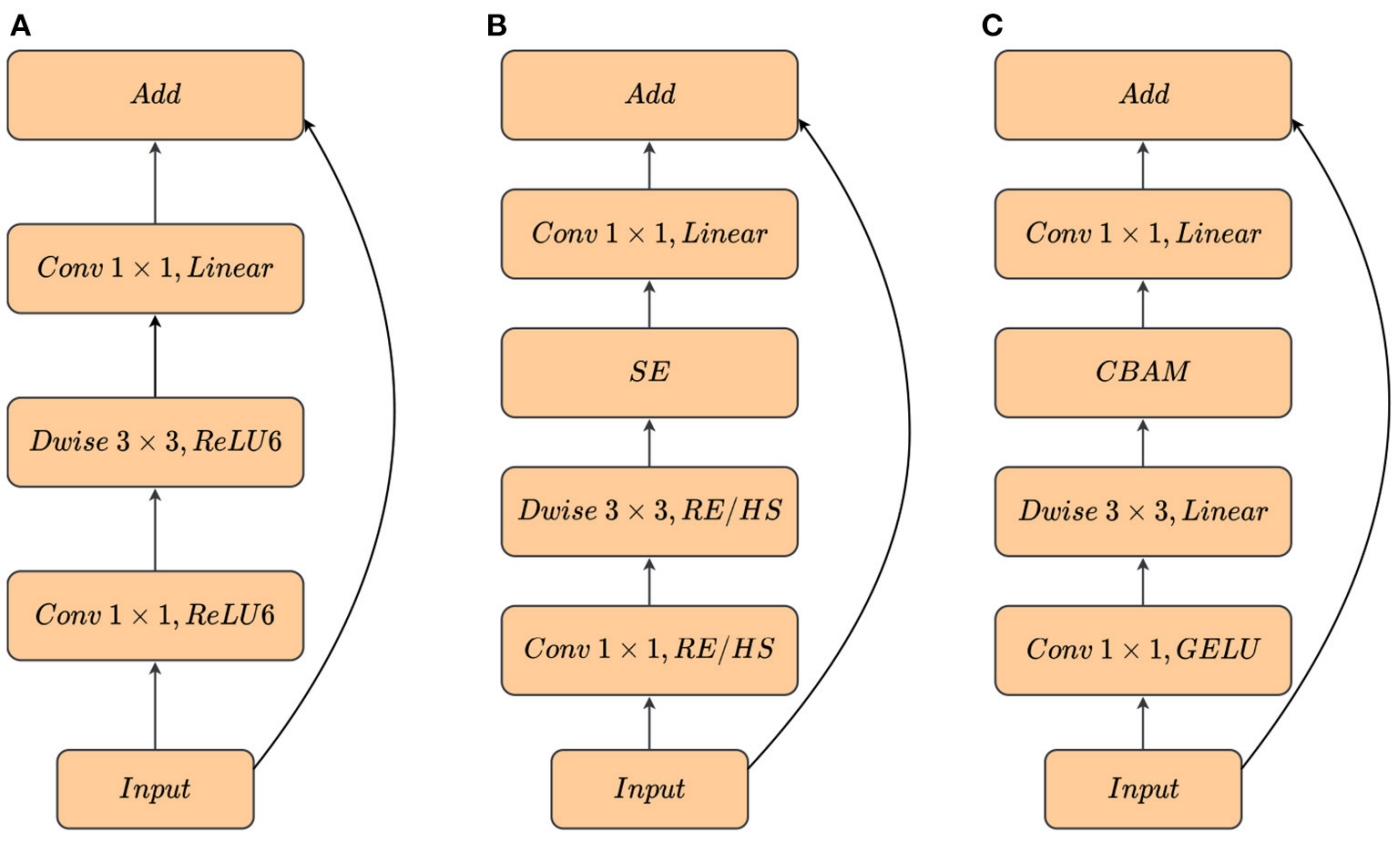
Comparison of different inverted bottleneck blocks. **(A)** MobileNetV2 block. **(B)** MobileNetV3 block. RE/HS denotes ReLU or h-swish. MobileNetV3 uses different activation function at different depths of the network. **(C)** MBConvA block. RE/HS with GELU is replaced in the first Conv and RE/HS is removed in Dwise. Linear indicates that there is no activation function.

Furthermore, we removed the activation function after DWconv, which improved *mAP* by 0.5%. The inverted bottleneck module applied to our mobile-end network was the Mobile Inverted Residual Bottleneck Convolution with Attention (MBConvA). The difference between the MobileNet block and our block is shown in Figure 5.

**Figure 6:**
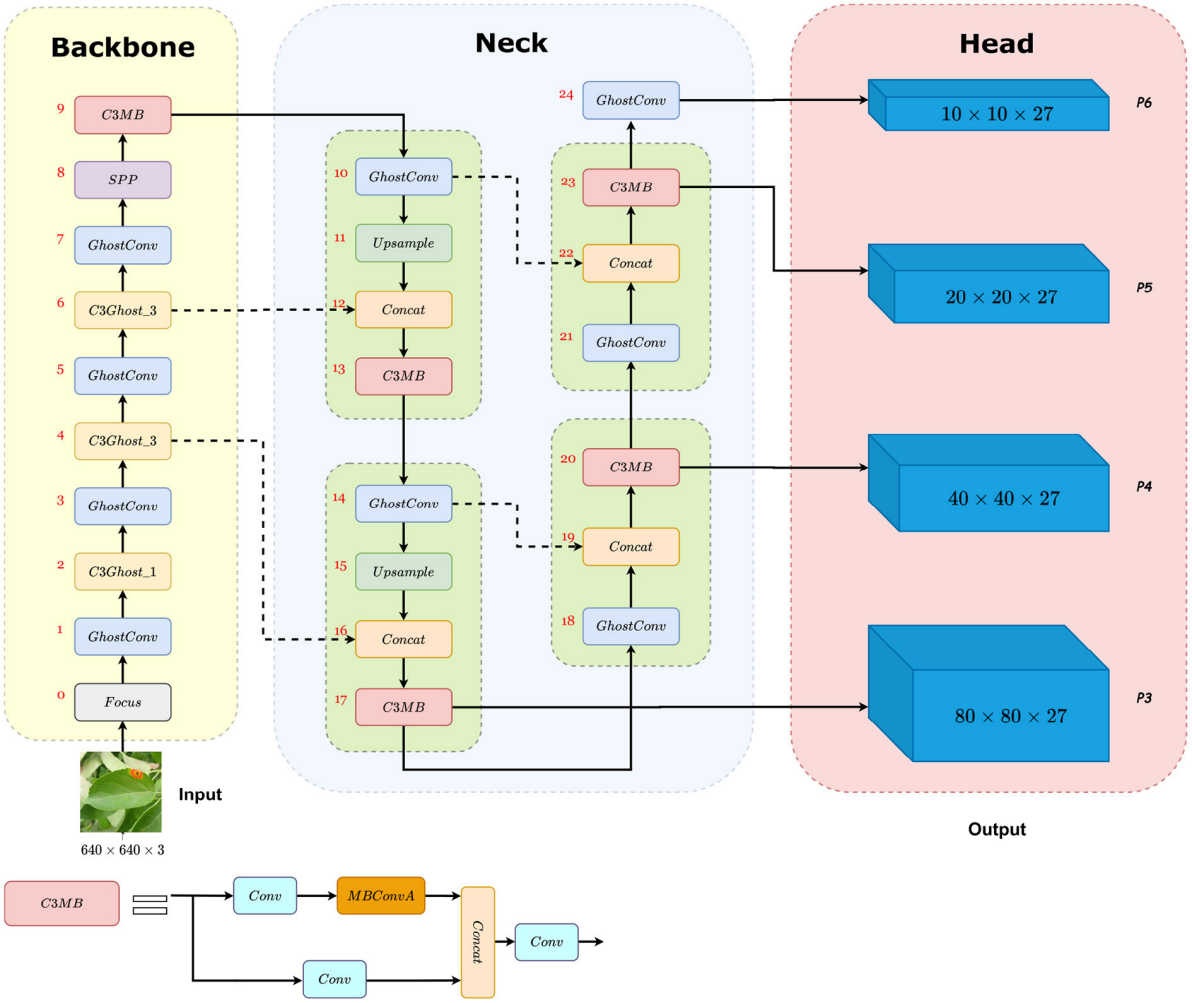
The architecture of the MGA-YOLO network. **Backbone**: CSPDarknet with Focus, GhostConv (the Ghost module), C3Ghost_X, and SPP. **Neck**: PANet with C3MB. **Head**: four prediction heads P3, P4, P5, P6 detect small, medium, large, and extra large objects, respectively. Firstly, 640 × 640 RGB images are given as the input, then the image features are extracted and fused through Backbone and Neck. Finally, four prediction heads with four different sizes and 27 channels are the output. Each prediction head corresponds to three anchor boxes and each anchor box predicts the probability of four categories, four attributes of the predicted bounding box (*x, y, w, h*) and the confidence of the predicted result. Therefore, the number of prediction head channels is 3 × (4 + 4 + 1) = 27.

We replaced the Bottleneck module with the MBConvA module in the C3_X module. In YOLOv5s architecture, *X* is approximately equivalent to 1, so each C3_1 contains one MBConvA module. We name the new block C3MB, which is shown in Figure 6.

### 3.5. The MGA-YOLO network

#### 3.5.1. Adding a detection layer

In the backbone network, we used the C3Ghost module to extract features. In the neck network, the C3MB module was used to focus on interesting objects and extract useful semantic information. Downsampling was done using the Ghost module with a stride of 2, along with the whole network. In addition, we examined the ALDOD dataset and found that most images contained extra large leaf targets, so we added one more prediction head to detect extra large objects. Compared with YOLOv5s-P6 models, we simplified the process of adding the detection layer. Based on the YOLOv5-P5 neck network, we directly added a 1 × 1 Ghost module with a stride of 2 after the P5 head, as the P6 prediction head. This method can effectively improve the detection capability of extra large targets without changing the depth of PANet, thus significantly reducing the number of model parameters and FLOPs compared with YOLOv5s-P6.

#### 3.5.2. Replacing ReLU with GELU

In YOLOv5 v5.0, the non-linearity Swish (Ramachandran et al., 2017) substitutes for the ReLU activation function. What distinguishes the two non-linearities is the continuous non-monotonic bump of Swish. Although ReLU and Swish are very similar in shapes, the Swish curve is smooth when the domain of definition is around zero. It does not abruptly change direction as ReLU does near *x* = 0. Instead, it smoothly bends from zero toward values < 0 and then goes upwards. Due to its non-monotonic bump, those negative values that could be relevant for capturing patterns underlying the data are retained, which significantly improves the accuracy of neural networks. The Swish non-linearity is defined as:


(5) $$\begin{array}{r} Swish\left(x\right)=x\cdot sigmoid\left(\beta x\right)=\frac{x}{1+e^{-\beta x}} \end{array}$$

YOLOv5 v5.0 uses Swish with a fixed *β* = 1, which is called Swish-1 (also known as SiLU), as the activation function.

**Figure 7:**
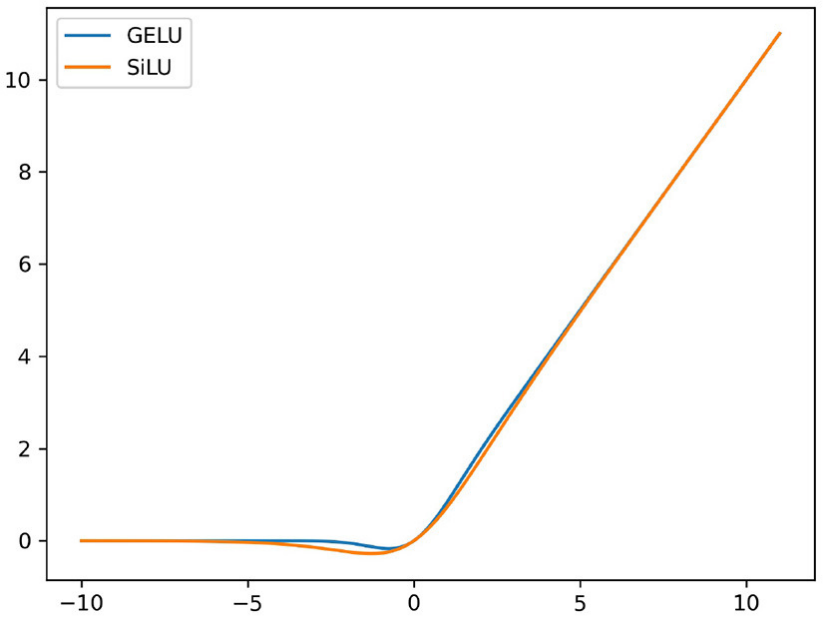
Comparison of GELU and Swish-1 curves. Both are non-monotonic functions and have similar shapes. When the input values are around zero, most outputs from GELU are greater than Swish-1 which makes the non-monotonic bump of Swish-1 wider than the other.

The Gaussian Error Linear Unit, or GELU (Hendrycks and Gimpel, 2020), which can be regarded as a smooth counterpart of ReLU, also has the non-monotonic bump similar to Swish-1. The GELU non-linearity is defined as:


(6) $$\begin{array}{r} GELU\left(x\right)=x\cdot \phi \left(x\right)=x\cdot \frac{1}{2}\left[1+erf\left(\frac{x}{\sqrt{2}}\right)\right] \end{array}$$

where ***ϕ***(*x*) is the standard Gaussian cumulative distribution function. GELU can approximate *x* · *sigmoid*(1.702*x*) so the difference between GELU and Swish-1 lies in the different value of *β*. The curve of Swish-1 and GELU are shown in Figure 7.

Currently, mainstream Transformers, including Google's BERT (Devlin et al., 2019), OpenAI's GPT-2 (Radford et al., 2019), and ViTs (Kim et al., 2021), utilize the GELU activation function as the non-linearity function. Our experiments found that GELU achieved a promising increase on *mAP* while the FLOPs remained unchanged. Thus, we substituted GELU for Swish-1 in our network.

Generally, we updated the original YOLOv5s-P5 to MGA-YOLO to improve leaf detection performance on ALDOD. The structure of MGA-YOLO is shown in Figure 6.

## 4. Experiments

This section describes the experimental setup and the dataset ALDOD in detail. We compared the performance of MGA-YOLO and other state-of-the-art object detection methods. In addition, an experiment was conducted to investigate appropriate image augmentation methods. We also conducted an ablation experiment to explore the effect of each proposed technique and analyze the results.

### 4.1. Experimental setup

Experiments were conducted on a high-performance deep learning server, which was equipped with two Nvidia RTX A4000 graphics cards with 16 GB graphics memory each. The operating system was Ubuntu 21.04 (64-bit). The implementation of the proposed method was based on Pytorch 1.7.1. The details of the experimental setup are given in Table 1. We also deployed our model on a HUAWEI Mate 40 Pro (4 G) mobile phone, with the HiSilicon Kirin 9000 CPU and the Harmony OS 2.0.0 operating system, to test the performance of our model.

**Table 1:** Details of the experimental setup.

**Item**	**Specification**
Central processing unit	Intel Xeon Gold 5218R CPU @ 2.10 GHz
Graphics processing unit	Nvidia RTX A4000 16 GB × 2
Memory	126 GB
Hard disk drive storage space	51 GB
Operating system	Ubuntu 21.04 (64-bit)
Programming environment	Python 3.8.8, Cuda 10.1, torch 1.7.1, torchvision 0.8.2, torchaudio 0.7.2

To optimize network parameters, MGA-YOLO utilized stochastic gradient descent (SGD) for training. We set the dynamic learning rate to accelerate the model convergence and maintain training stability. The initial learning rate (*lr*0) was set to 0.01, and the final OneCycleLR learning rate (*lrf*) was set to 0.2 to update the learning rate of each epoch. Given the current epoch *x*, we needed an intermediate variable *lf* as the multiplier for the learning rate. The learning rate (*lr*) for each epoch was updated as:


(7) $$\begin{array}{r} lf=\frac{1\;-\;\cos\left(\frac{x}{epochs}\cdot x\right)}{2}\times \left(lrf-1\right)+1 \end{array}$$

(8) $$\begin{array}{r} lr=lr\times lf \end{array}$$

Consequently, the final learning rate was *lr*0 × *lrf*.

### 4.2. The apple leaf disease object detection dataset

**Figure 8:**
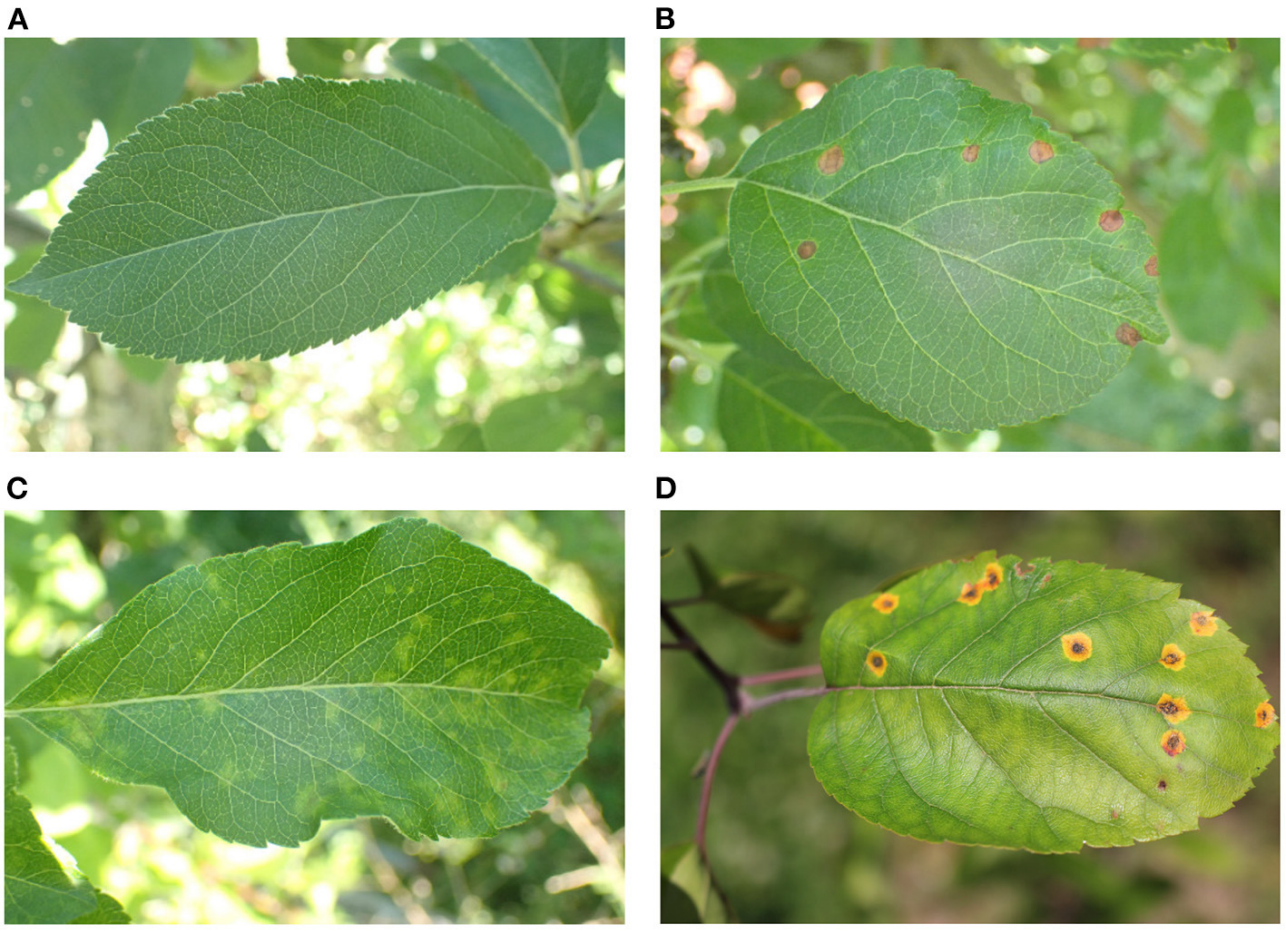
Representative images of healthy, rust, scab, and black rot apple leaves. **(A)** Healthy. **(B)** Rust. On leaves, cedar-apple rust first appears as small, pale yellow spots on the upper surfaces. The spots enlarge and eventually tiny black fruiting bodies become visible. Often several orange-yellow protuberances are produced in each spot on the underside of the leaf. **(C)** Scab. The scab leaf spots are round, olive-green, and up to 1/2-inch across. Spots are velvet-like with fringed borders and often form along the leaf veins. As they age, leaf spots turn dark brown to black, grow bigger, and join together. **(D)** Black rot. The fruit rot phase is called black rot (also called frog eye leaf spot) on leaves. At the beginning of frogeye spot disease, tiny purple specks appear on infected leaves. Gradually, they grow larger into a round spot with a light brown-to-gray center surrounded by one or more dark-brown concentric rings with a purple margin, giving it a “frog eye” appearance.

Apple leaf disease images in our dataset were collected from the public datasets Plant Pathology 2021-FGVC8 and Plant Pathology 2020-FGVC7 on Kaggle. The apple leaf images were divided into four categories, which included healthy leaves and three types of common leaf diseases: rust, scab, and black rot. Since the majority of the images has a resolution of 4, 000 × 2, 672 pixels, the details of apple leaves are preserved while backgrounds are influenced by shadows and occlusions with complex lighting conditions. This imitates the real application scenarios and potentially enhances the robustness of the trained model. The characteristics of the three apple leaf diseases are significantly different. Figure 8 shows the representative images of the four categories.

**Figure 9:**
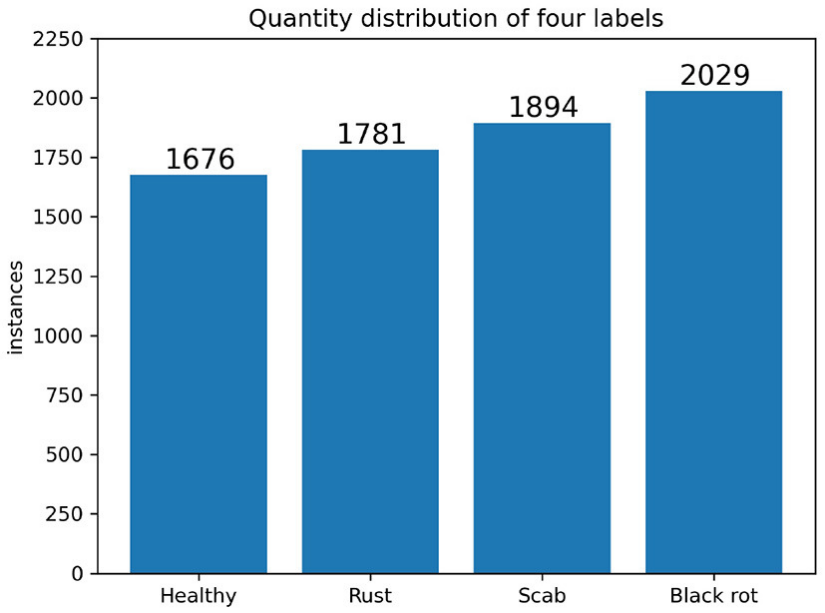
Quantity distribution of four labels of the training and validation datasets. The numbers of healthy, rust, scab, and black rot labels are 1,676, 1,781, 1,894, and 2,029, respectively. The total number of images is 6,802 and the total number of labeled leaves is 7,380, which means that there are 7,380 leaf objects for training and validation.

Image annotation is crucial for building the dataset. The original images from the two public datasets only had class labels for each image, but our method aims to detect each leaf in every image with a class label. Thus, we cannot directly use the original image datasets for training, validation, and testing. We used the annotation tool labeling based on Python to label each leaf in every image with a bounding box and a class label. We annotated entire leaves and drew the smallest circumscribed rectangle of each focused and unobstructed leaf during our labeling process. The number of images in each category is approximately the same to balance the distribution of different labels. It ensured balanced sample distribution and avoided over-fitting caused by the skewness of a specific class. Figure 9 shows the number of labels of four categories on the ALDOD training and validation set. In addition to the data formats for training they YOLO network, we also had annotations in PASCAL VOC (Everingham et al., 2010) and MS COCO (Lin et al., 2014) formats to facilitate the comparison with other state-of-the-art (SOTA) object detection methods. ALDOD has 8,838 images. The training dataset, validation dataset, and test dataset were divided in a ratio of 0.54:0.23:0.23, which correspond to 4,766, 2,036, and 2,036 images in each set, respectively.

Note that each leaf in our dataset ALDOD was labeled with a class of a specific disease. When multiple diseases occur on the same leaf, our model selects the class with the highest predicted probability as the prediction result. The public dataset Plant Pathology 2021-FGVC8 also provides images of multiple diseases on the same leaf. For future work, we plan to incorporate these images into our dataset and train a model that can recognize multiple diseases on the same leaf.

### 4.3. Image augmentation

To adapt MGA-YOLO to different environmental conditions and reduce the negative impact of photometric distortion (Zhu et al., 2021), the dataset was first expanded by random HSV adjustments, translations, shearing, rotating, scaling, and horizontal flipping. In addition to traditional data augmentation technologies, the Mosaic (Bochkovskiy et al., 2020) method is widely used in one-stage detection algorithms. Mosaic combines four training images to one in specific ratios. This enriches the background information of detected objects significantly. Based on previous image augmentation techniques, we investigated the effects of Mosaic. Based on the experimental results (see Section 4.6.6 for details), we used Mosaic and other techniques mentioned above.

### 4.4. Evaluation metrics

We utilized the following criteria to evaluate the performance of the model quantitatively.


(9) $$\begin{array}{rcl} Precision & = & \frac{TP}{TP\;+\;FP} \end{array}$$

(10) $$\begin{array}{rcl} Recall & = & \frac{TP}{TP\;+\;FN} \end{array}$$

*Precision* is a measure of result relevancy, while *Recall* measures how many truly relevant results are returned. The number of successfully recognized objects is represented by *TP* (True Positive), the number of incorrectly detected objects is represented by *FP* (False Positive), and the number of missed objects is represented by *FN* (False Negative). Furthermore, our model should be comprehensively evaluated in terms of detected boundaries and classification performance. The most widely used criterion is the Mean Average Precision, or *mAP*, employed in the following tests. In addition, *mAP* needs to be evaluated with a threshold *IoU*.


(11) $$\begin{array}{r} IoU\left(m,n\right)=\frac{area\left(m\;\cap \;n\right)}{area\left(m\;\cup \;n\right)} \end{array}$$

where *m* represents the ground-truth box and *n* represents the bounding box. *AP*_50_ and *mAP* are applied to evaluate the overall performance of detection. *AP*_50_ represents the average precision value when the threshold is set to 0.5 while *mAP* refers to the mean average precision values at different *IoU* thresholds ranging from 0.5 to 0.95, with a stride of 0.05.

### 4.5. Performance of the MGA-YOLO network

**Figure 10:**
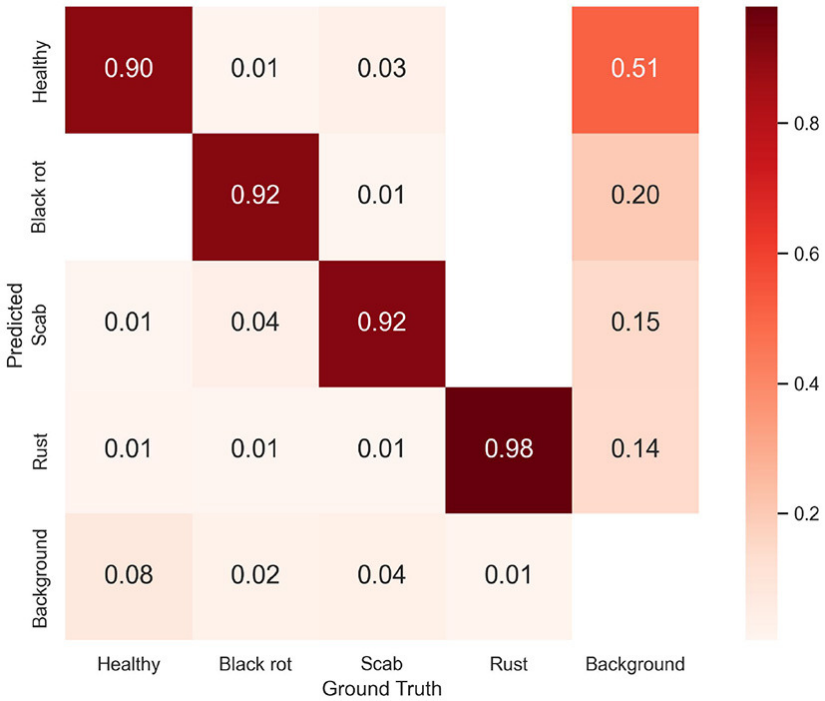
The confusion matrix of identification results. The confusion matrix was normalized over the true (columns) condition for our proposed model. The horizontal axis represents the ground truth classes and the vertical axis represents the predicted classes. Each cell element represents the proportion of the number of the predicted class to the total number of the true class. The diagonal elements represent correctly classified outcomes. All other off-diagonal elements along a column are wrong predictions.

**Figure 11:**
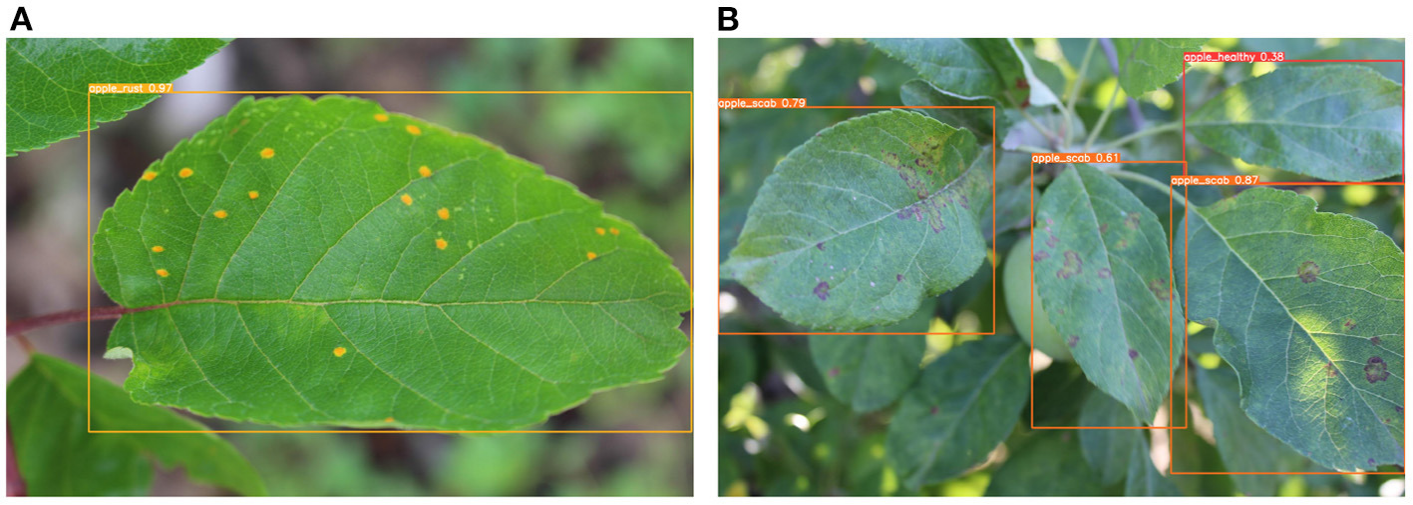
Examples of apple leaf disease detected by MGA-YOLO. **(A)** A leaf infected with rust is detected. **(B)** Three leaves infected with scab and a healthy leaf are detected. Each predicted bounding box shows the predicted label of the detected leaf and the confidence of the predicted result.

On the testing set with 2,036 images, the MGA-YOLO network accurately identified three apple leaf diseases and healthy leaves, with *AP*_50_ reaching 96.7% and *mAP* reaching 94.0%. The detection performance of each category is shown in Table 2 and the confusion matrix of detection results is shown in Figure 10. Figure 11 shows two examples of the detected apple leaf diseases.

**Table 2:** The detection performance of each category.

**Category**	**Labels**	**Precision**	**Recall**	**AP_**50**_**	**mAP**
All	2,193	0.955	0.908	0.967	0.940
Healthy	508	0.907	0.856	0.943	0.919
Black rot	603	0.991	0.912	0.977	0.958
Scab	575	0.950	0.901	0.960	0.938
Rust	507	0.970	0.964	0.987	0.945

We comprehensively evaluated the accuracy, the detection speed, and the weight of the model using metrics, including *mAP*, *AP*_50_, model size, and FPS. To demonstrate the superior performance of our model architecture, we compared our proposed MGA-YOLO method with several SOTA object detection algorithms. Table 3 compared the performance of two two-stage methods and six one-stage methods. Note that in this part of the experiments, we did not use Mosaic image augmentation on the training dataset to eliminate the influence of other factors on accuracy.

**Table 3:** Comparison of SOTA models.

	**Backbone**	**mAP(%)**	**AP_**50**_(%)**	**Weight(MB)**	**FPS**
**Two-stage methods**					
Faster-RCNN	ResNet50+FPN	87.4	94.4	322.69	8.19
Cascade-RCNN	ResNet50+FPN	87.9	**94.8**	540.13	7.32
**One-stage methods**					
SSD512	VGG16	84.4	93.6	194.04	15.12
RetinaNet	ResNet50+FPN	86.3	94.1	251.73	22.36
FCOS	ResNet50+FPN	84.7	91.5	250.26	8.29
YOLOv5X	CSPDarknet	87.7	92.3	171.01	31.63
YOLOX-L	CSPDarknet	88	94.7	635.82	15.05
MGA-YOLO	CSPDarknet	**89.3**	**94.8**	**10.34**	**84.13**

Best values are in bold.

We conclude that MGA-YOLO obtains the highest *mAP* of 89.3% on the ALDOD testing set, which is 1.3% higher than the second-place YOLOX_L. *AP*_50_ is also the highest, on par with Cascade-RCNN. Our model has the best recognition accuracy and achieves the fastest inference speed and the smallest model size compared to all the SOTA methods. The FPS of 84.1 on the GPU server and the model size of 10.34 MB meet the requirements for real-time object detection on embedded mobile devices.

Finally, we tested the performance of our MGA-YOLO network on a HUAWEI Mate 40 Pro mobile phone. Based on the Kirin 9000 CPU of this mobile phone, MGA-YOLO achieved 12.5 FPS for real-time detection, given images with a resolution of 256 × 256 as input, without Cuda GPU support.

### 4.6. Ablation experiments and analyses

An ablation experiment was conducted on the ALDOD testing set to investigate the effects of the modules in MGA-YOLO (see Table 4 for results). We took the YOLOv5s model as the baseline and tested with the Mosaic augmentation and other traditional techniques in the ablation experiment.

**Table 4:** Results of the ablation experiment on the ALDOD testing set.

**Model**	**mAP(%)**	**AP50(%)**	**Parameters**	**GFLOPs**	**Weight(MB)**
YOLOv5s	90.3	95.6	7,071,633	16.4	14.064
YOLOv5s-ghost	91.1	95.2	**3,703,993**	**8.2**	**7.598**
YOLOv5s-ghost-C3MB	92.2	95.8	5,679,145	11.3	11.466
YOLOv5s-ghost-C3MB-SE	91.0	95.8	8,708,233	11.4	17.391
YOLOv5s-ghost-C3MB-CA	91.0	95.6	6,256,381	11.4	12.617
YOLOv5s-ghost-C3MB-CBAM	92.5	96.0	6,440,256	11.4	12.946
YOLOv5s-ghost-C3MB-CBAM-Prediction_Head	93.6	96.6	7,641,179	11.6	9.654
YOLOv5s-ghost-C3MB-CBAM-Prediction_Head-GELU	**94.0**	**96.7**	7,641,179	11.6	10.337

YOLOv5s-ghost-C3MB-CBAM-Prediction_Head-GELU denotes MGA-YOLO. Best values are in bold.

#### 4.6.1. The ghost module

Generally, the Ghost module plays a vital role in reducing FLOPs and model size while maintaining accuracy. By replacing ordinary convolution and the C3_X module with the Ghost module and the C3Ghost_X module, respectively, in the backbone network, the parameters GFLOPs and model size had a sharp decline, which were almost halved. It can also be observed that *mAP* rose while there was only a slight decrease in *AP*_50_.

#### 4.6.2. C3MB

C3MB used in the neck network changed the parameters from 3.7 to 5.7 million, GFLOPs from 8.2 to 11.3, and model weight from 7.60 to 11.47 MB, but it has made a significant improvement on *mAP*. By applying the Ghost module and the C3 MB module to the network, *mAP* and *AP*_50_ are higher than the baseline, while the number of parameters, GFLOPs and model size are less than that of the baseline.

#### 4.6.3. CBAM

In this section, we compared three attention mechanisms and studied their effects. SE took the lead in extracting features in MobileNetV3. CBAM was integrated into YOLOv5 to increase drone detecting (Zhu et al., 2021). Recently, Coordinate Attention was proposed and used for apple leaf disease detection (Wang et al., 2021). The three attention modules above can be easily embedded into CNNs. It turned out that SE and CA cannot improve average precision but CBAM effectively improved *mAP* by 0.3% and *AP*_50_ by 0.2%. Meanwhile, there is not much computational overhead.

#### 4.6.4. The added prediction head

Adding a prediction head for extra large objects greatly contributed to the improvement of average precision. Although it brought about an increase in FLOPs by 0.2 G, the model size decreases from 12.9 to 9.7 MB. Experimental results showed that the inference speed was still fast.

#### 4.6.5. The GELU non-linearity

We substituted the GELU non-linearity for all the activation functions in the whole network. GELU did not change the parameters and FLOPs of our network, but surprisingly, it dramatically increased *mAP* by 0.6% resulting in 94%, showing that GELU was a better activation function than SiLU. Thus, the increase in the model size of 0.69 MB from 9.65 to 10.34 MB was acceptable.

#### 4.6.6. Mosaic image augmentation

With the traditional data augmentation technologies applied, MGA-YOLO had *mAP* of 89.3% and *AP*_50_ of 94.8%. Mosaic brought about a significant improvement to *mAP* by 4.7% and *AP*_50_ by 2.0% resulting in 94.0% and 96.7%, respectively, in the MGA-YOLO model. In addition, with Mosaic and other traditional image enhancement techniques, *mAP* of the baseline model reached 90.3% and *AP*_50_ reached 95.6%, both higher than MGA-YOLO without Mosaic. Therefore, the augmentation effect of Mosaic is significant on mAP and AP50.

#### 4.6.7. Overall analysis

Compared with the baseline YOLOv5s, the improvements described above for YOLOv5s brought about a significant increase in *mAP* from 90.3 to 94.0% and *AP*_50_ from 95.6 to 96.7%. Furthermore, the inference speed of the model has also been improved as FLOPs decreased from 16.4 to 11.6 G while the model size decreased from 14.1 to 10.34 MB.

Our Mobile Inverted Residual Bottleneck Convolution and Convolutional Block Attention Module enabled the baseline YOLOv5s model to have more vital feature learning capability. The extra prediction head improved semantic feature extraction capability in higher-level spatial dimensions. The Ghost module significantly reduced the number of parameters and FLOPs due to its effective cheap operation. GELU added better non-linearity to YOLOv5s. In general, the results of our experiment showed that the MGA-YOLO model outperformed the baseline.

In the ablation experiment, we used the Mosaic augmentation (Bochkovskiy et al., 2020; Jocher et al., 2021) method with other traditional augmentation techniques to improve the average precision of object detection. The detection results of MGA-YOLO in Tables 3, 4 shows that the image augmentation methods significantly improved *mAP* from 89.3 to 94.0% and *AP*_50_ from 94.8 to 96.7%. Traditional image augmentation techniques, including flipping, rotating, and shearing etc., enabled the image dataset to have more variants and avoid over-fitting. Moreover, the Mosaic image augmentation integrated all the data of four images into one image, which is equivalent to adding a series of objects with different scales for model training. It greatly enhanced the background information of the trained leaves. Due to these data augmentation techniques, the performance of our model was improved, as shown by *mAP* and *AP*_50_.

## 5. Discussion

Based on the architecture of YOLOv5, we introduced several effective modules that updated the original YOLO v5 architecture, which enabled it to accurately and efficiently identify healthy and infected leaves. However, questions that remain unanswered include how to strike a balance between accuracy and efficiency and how to choose the best model for a specific application scenario. In the task of identifying diseases, the priority is to extract fine-grained features and correctly identify the types of diseases. In this case, higher accuracy is needed and is more important than other factors. On the other hand, assuming that the objects we intend to recognize have highly recognizable features, and the task requires processing video streams in high speed, achieving a higher detection speed will be the top priority. In that case, we can sacrifice the feature extraction capability of the model in exchange for detection speed. As for the leaf disease detection task, in which accuracy was the most important factor, the proposed MGA-YOLO is more suited than other models.

In the present study, we tested the performance of the model on a CPU-based mobile phone. However, in orchards, environmental data are often collected through sensors, prepared by edge-computing devices and transferred to cloud servers for further data analyses. Obviously, a smartphone-based application for leaf disease detection is not sufficient for 24-h monitoring of orchards. Rapid development of AI and IoT devices and infrastructure has merged these two transformative technologies into Artificial Intelligence of Things (AIoT). Chen et al. (2020) constructed an AIoT-based pest detection smart agricultural system. Wireless transmission vision sensor modules were evenly arranged on the hillside of an orchard for continuous data collection, the Raspberry Pi was utilized to aggregate and upload the collected data to the cloud database to provide farmers with real-time observations of environmental changes. Our proposed model can be integrated into a system like this to enable continuous monitoring of apple leaves.

In addition to mobile phones, Raspberry Pi (Park et al., 2017) is widely used in smart agriculture as an edge computing module. Therefore, for future research, we intend to conduct comparisons with other SOTA methods on the Raspberry Pi platform to further evaluate the performance of the model.

Recently, some high-performance edge computing modules or small AI supercomputers, such as Nvidia's Jetson series, have been introduced to agriculture engineering (Guillén et al., 2021). These edge computing modules often support GPU acceleration and are affordable. Based on such platforms, deep learning networks can achieve better performance compared to CPU-based mobile platforms. It can be expected that GPU-enabled edge computing devices will be more widely used in agricultural engineering in the near future.

## 6. Conclusion

In this paper, we proposed a lightweight one-stage convolutional neural network, called MGA-YOLO, for real-time apple leaf disease detection. To evaluate our proposed method, we collected 8,838 apple leaf images of four categories from public datasets to build the ALDOD dataset with manual annotation. We used various image augmentation techniques to augment the dataset for apple leaf disease detection. The Ghost module, CBAM and other effective techniques enabled MGA-YOLO to perform better than other SOTA methods on the ALDOD testing set, with the highest average precision, the fastest detection speed and the smallest model size. The proposed model adopted many techniques to balance accuracy and efficiency for the apple disease detection task. Since the diagnosis methods of leaf diseases of different plants are similar, our method can also be applied to disease diagnosis of other crop or fruit leaves, making it have a wider range of applications.

## Data availability statement

The datasets presented in this study can be found in online repositories. The names of the repository/repositories and accession number(s) can be found at: www.kaggle.com/dataset/df248b05bcf0246cc0b7add831501d83a30396900302f3e6d01ab293471150f4.

## Author contributions

YiW and YaW collected data and designed the experiments. YiW conducted the experiments, analyzed the results, and wrote the first draft of the manuscript. YaW and JZ revised the manuscript. All authors have read and approved the final manuscript.

## Funding

This research was funded by the National Natural Science Foundation of China (72171005).

## Conflict of interest

The authors declare that the research was conducted in the absence of any commercial or financial relationships that could be construed as a potential conflict of interest.

## Publisher's note

All claims expressed in this article are solely those of the authors and do not necessarily represent those of their affiliated organizations, or those of the publisher, the editors and the reviewers. Any product that may be evaluated in this article, or claim that may be made by its manufacturer, is not guaranteed or endorsed by the publisher.
